# COVID-19 Era and the Constantly Reemerging Novel SARS-CoV-2 Variants Calls for Special Attention for the Geriatrics: A Real Challenge

**DOI:** 10.3390/geriatrics7060143

**Published:** 2022-12-19

**Authors:** L. V. Simhachalam Kutikuppala, Venkataramana Kandi, Ashish K. Sarangi, Snehasish Mishra, Ranjan K. Mohapatra

**Affiliations:** 1 Department of General Surgery, Dr. NTR University of Health Sciences, Vijayawada 520008, Andhra Pradesh, India; 2 Department of Microbiology, Prathima Institute of Medical Sciences, Karimnagar 505417, Telangana, India; 3 Department of Chemistry, School of Applied Sciences, Centurion University of Technology and Management, Balangir Campus, Parlakhemundi 767001, Odisha, India; 4 School of Biotechnology, Campus-11, KIIT Deemed-to-be-University, Bhubaneswar 751024, Odisha, India; 5 Department of Chemistry, Government College of Engineering, Keonjhar 758002, Odisha, India

Global public health is significantly challenged due to the continuing COrona VIrus Disease 2019 (COVID-19) outbreak brought forth by the severe Acute Respiratory Syndrome Corona Virus-2 (SARS-CoV-2) [1,2,3]. A highly contagious and deadly disease, COVID-19 caused more than 600 million confirmed cases and more than 6.5 million fatalities globally by October 2022 [4]. The COVID-19 pandemic has had a significant impact on the older adults. The geriatrics, who are often weak and have comorbid conditions, are significantly more susceptible to COVID-19 pandemic with higher risks of terrible morbidity and fatality rates [5]. Despite the fact that the global average mortality rate was 3.4%, it is estimated that the mortality rate in the age group 60 and 69 was 8.4%, and it was nearly four-fold in people over the age of 80 [6]. Increasing frailty, immuno-senescence, and chronic illnesses, which are seemingly characteristic to the ageing population, may reduce people’s resilience to COVID-19. It may make them more susceptible to social exclusion, leading to detrimental long-term effects on their physical and mental health. Frail older persons who are not physically active are more likely to be COVID-19 infected [7,8]. COVID-19-infected geriatrics experience a wide range of clinical manifestations from asymptomatic infection to catastrophic disease with multiple organ failure that may ultimately lead to death. The long-term consequences of COVID-19 infection in them often include disability, cognitive decline, and reduced immunity [9]. The spread of SARS-CoV-2 transmission was witnessed even in the face of lockdown and quarantine measures [10]. The geriatrics are urged to stay home to avoid contracting the virus due to an increased risk for seriously fatal illness.

Stringent disinfection measures may endanger their physical and psychological wellbeing, resulting in functional decline and poor quality of life, risking them long-term negative outcomes. Social isolation has affected their day-to-day normal life, forcing them to develop sedentary habits and move away from physically active life. As a result of fewer possibilities for either incidental or intentional physical activity, the skeletal muscle function could eventually be hampered and intrinsic capacity lost. Decreased levels of physical activity may lead to sarcopenia, a reduction in physical function, and poor quality of life [11]. Weakened immune system in the older generation makes them more vulnerable. SARS-CoV-2 is a novel coronavirus with no preceding immune response, making it unfeasible for senescent immune activities to counter it, thereby making the elderly population more vulnerable. The amount of peripheral blood lymphocytes normally drops over time, which generally indicates the severity and unfavourable outcomes of COVID-19 infection [12]. Data obtained over time showed that the lymphocyte counts were noticeably lower in the COVID-19-infected geriatrics. Moreover, SARS-CoV-2 mostly causes respiratory infections that exacerbates or precipitates allied clinical symptoms that make the infection control measures more challenging [13]. The antigen-response ability along with the intensity of antibody response in the geriatrics effectively decreases. As a result of both the lack of immunocompetence and age-dependent immuno-abnormalities, their response to vaccination is poor [14].

The elderly suffer from morbid conditions for an extended period after recovering from the clinical infection caused by SARS-CoV-2, a condition termed variably as post-COVID syndrome, long COVID, chronic COVID, post-acute COVID-19 syndrome, or post-acute sequelae of SARS-CoV-2 infection (https://www.idsociety.org/covid-19-real-time-learning-network/disease-manifestations--complications/post-covid-syndrome/; accessed on 8 November 2022). COVID-19-related social distancing and lockdowns had significant effects on the psychological wellbeing of the elderly that appears to continue despite vaccinations, indirectly compromising their overall health [15]. SARS-CoV-2 is a tough nut to crack considering its continued unpredictability as it evolves, evidenced by the emergence of variants that potentially evade immune responses and the vaccine-induced immunity. Moreover, it has recently been responsible for serious post-infection complications that severely affect the elderly and younger individuals. COVID-19 is a complex disease that invariably manifests its effects even after the recovery. The post-infection effects among the geriatrics are ill-understood as of yet. A recent study proposed to formulate geriatrics-specific guidelines to addresses recovery-stage complications in the elderly [16]. Studies have revealed that the elderly, especially those people who had a severe bout, show at least one post-COVID-19 complication even after two months of infection and needed hospitalisation with intensive care treatment. Morbid conditions identified in such cases included polyneuropathy, myopathy, post-intensive care syndrome, post-traumatic stress disorder, persistent fatigue, dyspnoea, and arthritis [17,18]. Another study noted that around 10% of geriatrics might show post-COVID-19 morbid conditions even after 90 days of recovery. Severe fatigue, chest discomfiture, cough, breathlessness, dizziness, anosmia, and ageusia persisted even after three months of recovery [19].

Interestingly, geriatrics suffering from clinically mild infections needing no hospitalisation then were also affected by post-COVID-19 issues like persistent post-viral fatigue syndrome [20]. Evidence-based patient management including a comprehensive approach to address health issues among post-COVID geriatrics was recently suggested. Post-COVID issues to address that were identified included the requirement of extramural oxygen, an extended thromboprophylaxis, screening for neurological problems like sleep disorders, and anxiety, among others [21]. It is suggested that post COVID-19 patients who suffered from acute kidney injury may opt for regular follow-up by a nephrologist. The elderly who were potentially predisposed to post-COVID syndrome must be recruited into rehabilitation programmes for careful assessment and management of organ specific-disorders. Patient advocacy groups, social media, and related platforms wherein the patients themselves form a group that regularly analyse the post-COVID-19 effects are suggested [21]. Since the geriatrics could already be suffering from comorbid conditions, geriatric rehabilitation special interest group of the European Geriatric Medicine Society (EuGMS) suggested that a multi-expert team that included geriatric physicians and other medical specialists was required to address the issues better and improve the quality of life of the elderly [22,23].

Older people are generally prone to develop drug-induced nutritional problems. Studies show the association between an increasing number of medications and malnutrition. It is also observed that COVID-19 has challenged the older adults to access food, particularly among the lower income and rural communities [24]. Food insecurity, low protein, and low fibre intake are significantly associated with the psychological distress among the old adults during the COVID-19 pandemic [25]. The impact of food insecurity, diet quality, and poor nutrient intake on psychological health is yet to be fully investigated. Implementing advanced food security programs and specific public policies for the elderly is suggested. More investigations to examine the impact of COVID-19 on food security and the differential barriers experienced by old adults are needed. Age is an important risk factor in the COVID-19 era, so proper nutrient intake might help in maintaining health and reducing the severity of the disease, particularly among geriatrics [26].

Strengthening the primary healthcare facilities that are considered as the point of first contact for patients seeking medical care was suggested as a method for improved care of the elderly [27]. Since the elderly are particularly susceptible to COVID-19, measures to control the infections and to minimise the morbidity and mortality assume great significance. Moreover, the geriatrics is a special group that should be encouraged to volunteer for clinical research aimed to understand the post-COVID syndromes, the effects of vaccination on SARS-CoV-2 infection, and the long-term effects in them [28]. Improvement of digital literacy, access to the internet, and community-based interventions could increase their awareness about the effects of SARS-CoV-2 infection and associated long-term sequelae [29]. A decline in the health-related quality of life (HR-QoL) six months after being discharged from hospital was recently observed among Norwegian patients above 60 years [30]. Improved knowledge and awareness of the long-term effects of COVID-19 was suggested to improve the HR-QoL. The elderly must be familiarised and educated about the potential post-COVID-19 signs and symptoms. Effective rehabilitation strategies that include home-based healthcare could notably improve the psychosocial behaviour and overall health of the elderly [31]. Caretakers with the necessary skills can offer vital tips through home-care to the vulnerable elderly during the pandemic. To ensure continued care of the elderly, investigating novel strategies like integrating healthcare facilities and hospitals into the health-care services through the IoT approach is crucial. Due to the uncertain future of the pandemic, efficient plans and tactics to aid in preventing COVID-19 risks and addressing geriatrics’ health are needed.
